# Income, Wealth and Health Inequalities — A Scottish Social Justice Perspective

**DOI:** 10.3934/publichealth.2016.2.255

**Published:** 2016-04-27

**Authors:** Elspeth Molony, Christine Duncan

**Affiliations:** NHS Health Scotland

**Keywords:** social justice, health inequality, income inequality, wealth, fairness

## Abstract

This paper considers health inequalities through a social justice perspective. The authors draw on a variety of existing sources of evidence, including experiential, scientific and contextual knowledge. The authors work with NHS Health Scotland, a national Health Board working to reduce health inequalities and improve health. Working closely with the Scottish Government and with a variety of stakeholders across different sectors, NHS Health Scotland's vision for a fairer, healthier Scotland is founded on the principles of social justice. The paper takes social justice as the starting point and explores what it means for two interlinked paradigms of social injustice—health inequality and income inequality. Utilising the wealth of evidence synthesised by NHS Health Scotland as well as drawing on the writings and evidence of philosophers, epidemiologists, the Scottish Government and international bodies, the authors explore the links between income and wealth inequality, social justice, the right to health and health inequalities. The paper ends by considering the extent to which there is appetite for social change in Scotland by considering the attitudes of the people of Scotland and of Britain to poverty, inequality and welfare.

## 1. Introduction

Income inequality in Scotland and the UK has been rising since the early 1980s [1]. There is compelling evidence that income inequality causes shorter, unhealthier and unhappier lives [2],[3]. In this article we consider income and health inequalities as issues of social justice and explore the public's attitude towards the creation of a fairer and healthier Scotland.

## 2. Social Justice

In his highly influential work *A Theory of Justice* [4], the political and social philosopher John Rawls presented the idea of justice as fairness—the idea that social institutions should not confer morally arbitrary lifelong advantages on some people at the expense of others. Rawls writes of the principles of social justice that:

“*…they provide a way of assigning rights and duties in the basic institutions of society and they define the appropriate distribution of benefits and burdens of social co-operation.*”

The United Nations defines social justice as “…*the fair and compassionate distribution of the fruits of* economic *growth*...” [5] and at NHS Health Scotland we have defined our understanding of social justice as “the fair and equal distribution of wealth, opportunities and privileges within society.” [6].

Social justice is a complex concept, with numerous debates abounding around the precise nature of fair distribution, and to what extent this may conflict with individual rights of ownership and acquisition. It is not our purpose here to enter this debate, but rather to take social justice as our starting point and explore what this means for health and income inequalities, two interlinked paradigms of social injustice.

## 3. Health Inequality

The overall health of the Scottish population is continuing to improve, along with a decline in the death rate [2]. However, the gaps between those with the best and worst health and wellbeing still persist, some are widening, and too many Scots still die prematurely. Health inequalities, the avoidable differences in people's health across social groups and between different population groups [7], represent thousands of unnecessary premature deaths every year in Scotland, and for men in the most deprived areas nearly 24 fewer years spent in ‘good health’ [8].

**Figure 1.**
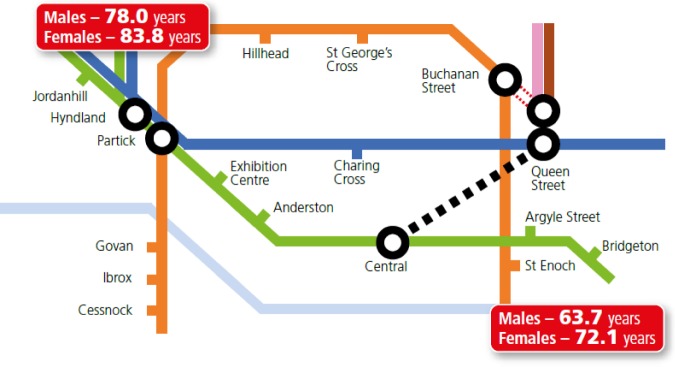
Life expectancy in Glasgow.

NHS Health Scotland has produced illustrations of the gap in life expectancy in Scotland's two main cities. Figure 1 below is based on the Glasgow trainline [9] and shows that life expectancy in men goes down by two years for every station on the trainline travelling from Jordanhill (in the west end) to Bridgeton (in the east end). On average, the life expectancy of a man born in Bridgeton is 14.3 years less than his counterpart in Jordanhill, and a woman 11.7 years less [10].

**Figure 2.**
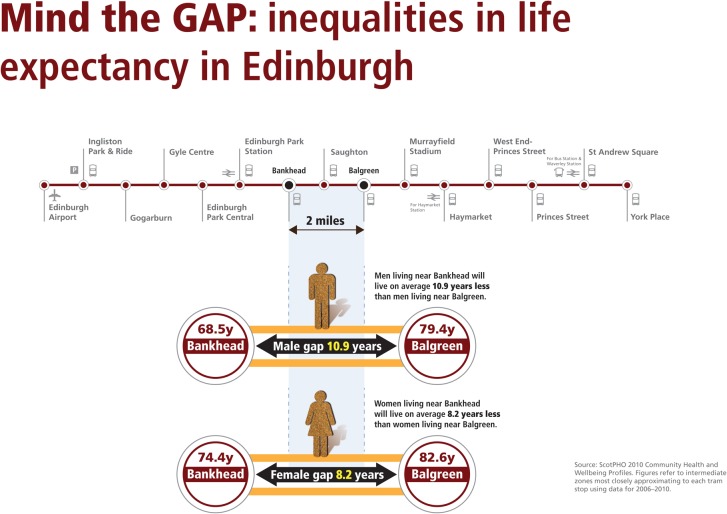
Life expectancy in Edinburgh.

In Edinburgh the extent of health inequalities is demonstrated by the difference in average life expectancy(Figure 2) for men and women living close to different stops on the city's tram line [11].

Health inequalities contravene the principles of social justice because they are avoidable; they do not occur randomly or by chance, but are socially determined by circumstances largely beyond an individual's control. These circumstances disadvantage people and limit their chance to live a longer, healthier life.

Health inequalities are rooted in political and social decisions that shape society and how it functions [12]. There was a substantial narrowing of health inequalities in the UK and USA between the 1920s and 1970s, the period in which welfare states were constructed and income inequalities declined [3],[13].

**Figure 3.**
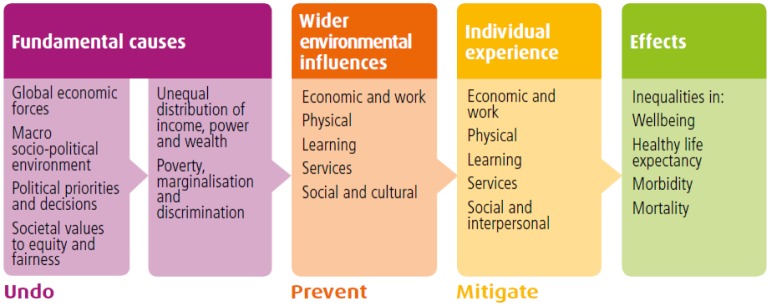
Health inequalities: theory of causation.

NHS Health Scotland conducted a policy review [3] in 2013 in order to provide information to the Scottish Ministerial Task Force on Health Inequalities. The review examined the causes of health inequalities and summarises the best available evidence about what can be done to reduce health inequalities. The review finds that “*The causal pathways which lead to health inequalities are numerous, interrelated and not fully understood*.” In order to articulate the current understanding of the causal pathways, NHS Health Scotland developed a theory of causation, which is set out in Figure 3 below.

Highlighting the findings of the Commission on Social Determinants of Health [14], set up by the World Health Organization (WHO) in 2005 to “marshal the evidence on what can be done to promote health equity”, the theory of causation shows that the fundamental causes of health inequalities are “the major sociopolitical forces which drive decisions and priorities and which, depending on the social and economic principles underpinning these decisions, result in an unequal distribution of power, money and resources.” [3].

The theory of causation goes on to show that these fundamental causes influence the distribution of wider environmental influences on health, such as the availability of good quality housing, work, education and learning opportunities, as well as access to services and social and cultural opportunities in an area and in society.

The wider environment in which people live and work then shapes their individual experiences of, for example, low income, poor housing, discrimination and access to health services.

## 4. The Right to Health

For some people, the injustice of health inequalities is experienced at an individual level as a barrier to the right to health [15], a right first articulated in the 1946 Constitution of the World Health Organisation (WHO) [16] and articulated in a number of international agreements since [17]. The WHO Constitution enshrines the highest attainable standard of health as a fundamental right of every human being. The right to health means that WHO member states must generate conditions in which everyone can be as healthy as possible, it does not mean the right to be healthy.

Since the 1946 WHO Constitution, the right to health has been articulated in a number of international agreements. The Universal Declaration of Human Rights, Article 25 (1) states that “*everyone has the right to a standard of living adequate for the health and well-being of himself and his family*” [18]. In addition, the International Covenant on Economic, Social and Cultural Rights [19], Article 12 guarantees the right of everyone to the enjoyment of the highest attainable standard of physical and mental health.

The right to health extends not only to timely and appropriate health care but also to the underlying determinants of health, such as access to safe drinking water and adequate sanitation, an adequate supply of safe food, nutrition and housing, healthy occupational and environmental conditions and access to health-related education and information [20]. In social justice terms—the fair and equal distribution of the conditions required for everyone to be as healthy as possible.

## 5. Wealth and Wellbeing

What is required to be as healthy as possible? We know that within societies, people with greater income and wealth are healthier [21], and various longitudinal studies have established that this relationship is largely causal: greater income and wealth leads to better health [22]–[24]. In order to maintain good health, individuals have to meet their core needs. This means having access to food, clean water, housing, leisure and various other goods and services, all of which come at a cost [3].

The word ‘wealth’ originates from the old English word ‘weal’ and means the abundance of valuable resources or valuable material possessions [25]. An individual, community, region or country that possesses an abundance of such possessions or resources to the benefit of the common good is considered to be wealthy. It is this concept of the common good that is critical to any discussion about wealth and its relationship to health inequality. In particular the relationship between economic growth or wealth creation and the common good. The pursuit of economic growth has been a key goal of western administrations in the latter 20th and into the 21st Century. Indeed the Scottish Government's purpose is described as:

*‘To focus Government and public services on creating a more successful country, with opportunities for all of Scotland to flourish, through increasing sustainable economic growth’* [26].

Central to this purpose is the principle of every citizen benefiting through opportunities to flourish, that wealth is shared as it is created so that everyone benefits. This is an aim based on principles of social justice as well as economic theory.

However there is evidence [27] that the benefits from wealth creation through economic growth are not shared equitably. The chart below, from the Scottish Government's recent summary of the statistics on income inequality [1] shows that wealth is unevenly distributed in Scotland. In the period 2010 to 2012, the least wealthy 40%of households (blue bars) in Scotland owned less than 5% of wealth; the ‘middle’ 50% (black bars) owned around half of wealth; and the wealthiest 10% of households (orange bars) owned 44% of wealth. The top 2% alone owned 20% of all personal wealth in Scotland.

**Chart 1.**
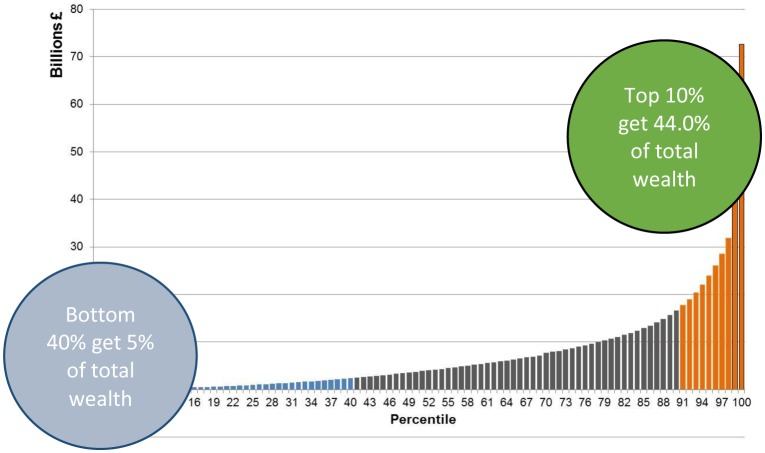
Wealth distribution in Scotland. (*Source: Scottish Government: Communities Analysis Division September 2015*)

Income is also unevenly distributed in Scotland. The chart below shows that the distribution of income is dominated by a small proportion of very high income households. In 2013/14, the bottom 40% of households (blue bars) had around a fifth of total household income; the ‘middle’ 50% (black bars) had just over half of all income. The top 10% of households (orange bars) had around a quarter of all household income—with the top 2% alone having nearly 10%.

There are two issues here; the negative impact poverty has on health and the negative impact income inequality has on the health of all citizens within a society.

The epidemiologist Professor Jerry Morris, prompted by the absence of health needs in the existing minimum income requirements, [28],[29] developed the Minimum Income for Healthy Living (MIHL). The MIHL describes the disposable income essential for health. It “includes not only what is necessary for food and shelter, but what is required to live a life of dignity and to take one's place

**Chart 2.**
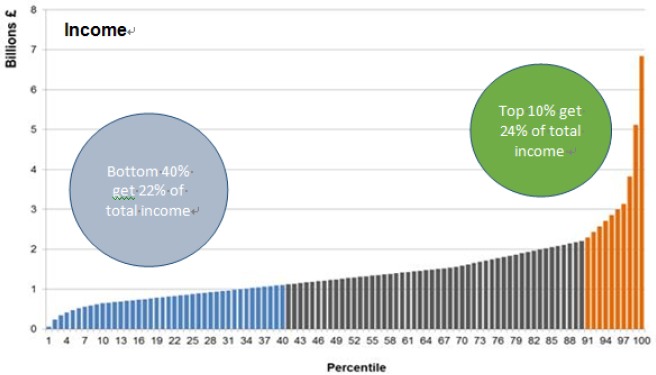
Income distribution in Scotland. (*Source: Scottish Government: Communities Analysis Division September 2015*)

There is evidence that increasingly paid employment and social security benefits do not provide a sufficient income for healthy living. In 2013/14, 430,000 people in Scotland were affected by in-work poverty after housing costs—more than half of all poor children and almost half of all poor working-age adults. [30] Poverty rates are higher for single working-age women than for single working-age men (29% vs. 26%), and especially high for female lone parents (31%), despite rising employment rates [31]. In Scotland, households who receive their main income from benefits are almost twice as likely to report they do not manage well financially as those in work (19% vs. 10%) [32].

Turning to the negative impact of income inequality, Wilkinson and Pickett argue in the Sprit Level: Why More Equal Societies Almost Always Do Better [2], that the less equal a society, the more *pernicious the effects of inequality.* The authors found that outcomes for eleven health and social problems (including physical and mental health, drug abuse, education, imprisonment, obesity, social mobility, trust, and community, violence, teenage pregnancy and child wellbeing) are significantly worse in more unequal countries. In short, inequalities of income damage the health and wellbeing of us all, wherever we find ourselves on the income or wealth spectrum.

As Michael Marmot says in The Health Gap [33] *“The gradient implies that the central issue is inequality, not simply poverty. Inequality… implies that not only is having enough to make ends meet important, but so too is what we have relative to others”*. It is not only those living in poverty, or those in the lower socioeconomic deciles who experience health inequalities—health inequality affects almost everyone, *“All the way, from top to bottom of society, the lower you are, the worse your health. The gradient includes all of us below the topmost 1 per cent.”*[34].

## 6. Appetite for Social Change?

We therefore have evidence that to create a fairer and healthier Scotland, which would benefit us all, we need to tackle the fundamental causes of inequality. That is, action is needed to redistribute income, wealth and power. The issue of what constitutes fair distribution of wealth, opportunities and privileges within society has been challenging philosophers since at least the time of Aristotle. The theories of redistribution and the mechanism by which it would be achieved are beyond the scope of this paper and indeed of NHS Health Scotland as an organisation. However, we would like to give consideration to the appetite for social change. Is the public ready and willing to support the required change?

We know from the Scottish Government research report ‘Public Attitudes to Poverty, Inequality and Welfare in Scotland and Britain’[34], which sets out public attitudes to poverty, inequality and welfare in Scotland and Britain, from a range of previously published sources, that there is a high level of concern about poverty and inequality and support for government action:

Nearly all people in Scotland (98%) felt it was important to tackle child poverty.

83% of people said that the gap between those on high incomes and those on low incomes was too large.

There was a high level of public agreement in 2012 about the ‘necessities of life’ everyone should have access to, including a heated and damp free home and two meals a day.

However, views on what causes poverty were mixed:

In 2010 in Britain, 23% of people thought that people live in need because of laziness or a lack of willpower, while 21% thought it was due to injustice in society.

Looking at child poverty specifically, 72% of people in Scotland in 2013 felt that this was caused by individual factors such as parents not wanting to work, with only 28% attributing it to structural factors such as inadequate social security payments. Parental alcoholism, drug abuse or other addiction was perceived as the most common main cause of child poverty in Scotland.

The ‘Necessities of Life’ survey [35], which was carried out between May and June 2012 and is based on a sample of 1,447 adults aged 16 or over in the Britain and 1,015 in Northern Ireland, found a more nuanced attitude towards those in poverty. As highlighted by the Scottish Government in ‘Public Attitudes to Poverty, Inequality and Welfare in Scotland and Britain’ [31], current economic and structural factors were seen as the greatest cause of poverty, although long-term structural causes and causes relating to individuals were also identified. There was a recognition that poor personal choices may be an outcome, rather than a cause, of poverty.

The views on the nature of interventions were mixed:

48% of respondents agreed that the government should redistribute income from the better off to the less well off, while 25% disagreed.

Almost half of people (48%) thought that taxes and public spending should be kept at the same level, while a large minority (44%) thought they should be increased. Only 4% thought that taxes and spending should be decreased.

In Britain as a whole, support for extra spending on benefits declined between the 1980s and 2011, but increased in 2012 and 2013.

## 7. Creating a Fairer and Healthier Scotland

The high level of concern about poverty and inequality and support for government action highlighted above is reinforced by the themes emerging from the Scottish Government's national conversations on *Creating a Fairer Scotland* [36] and *Creating a Healthier Scotland* [37]. These conversations are building on the civic engagement generated in the lead up to the Independence Referendum and asking the citizens of Scotland to get involved in shaping an action plan for social justice and for a health and social care system fit for the future. A number of personal stories of poverty and inequality have been shared through social media and through community events, stories that illustrate the reality of the issues touched on in this paper. Third sector organisations have also been lending their weight to the conversations.

In their blog, ‘*People with multiple sclerosis say the benefits system is harming their health*’, Morna Simpkins, Director of Multiple Sclerosis Society Scotland, said:

*“Scots living with MS are missing out on the support they need and having to make sacrifices many of us cannot imagine in order to get by. In our survey, a third told us that they have been forced to reduce spending on basic essentials as a result of benefit changes; 30% have reduced spending on food; 25% have cut down on gas and electricity and 47% have cut down on socialising with family and friends. This is unacceptable and people in Scotland with MS need the welfare system to change so that it makes sense.”* [38].

In the NHS Health Scotland blog ‘*Why a Fairer Scotland would be a Healthier Scotland*’ Gerry McLaughlin, Chief Executive of NHS Health Scotland said:

*“Improving the health of the population in an equitable way requires action across a range of public policy areas. For example, policies to tackle economic and social inequalities should happen alongside and at the same time as action to address the behavioural determinants of poor health…The focus needs to shift away from considering the problem as a responsibility to the specific individual, community or government towards the idea that this is everyone's responsibility. In the end, we are all affected and we all pay the cost. To create a fairer Scotland all of us need to strive towards action, to drive a fairer share of income, resources and power... The message is clear A Fairer Scotland will be a Healthier Scotland.”* [39].

The principles of fairness, equality and equity have emerged as key themes in both conversations, suggesting that there is a real willingness for change within civic society in Scotland. A willingness to support action to address the fundamental inequalities that are preventing A Fairer Healthier Scotland—a socially just Scotland—from becoming a reality.

*“Social justice is a matter of life and death. It affects the way people live, their consequent chance of illness, and their risk of premature death.”* [40].
